# Successful artificial insemination in the Asian elephant (*Elephas maximus*) using chilled and frozen-thawed semen

**DOI:** 10.1186/1477-7827-7-75

**Published:** 2009-07-19

**Authors:** Nikorn Thongtip, Sittidet Mahasawangkul, Chatchote Thitaram, Pornsawan Pongsopavijitr, Kornchai Kornkaewrat, Anuchai Pinyopummin, Taweepoke Angkawanish, Saran Jansittiwate, Ronnachit Rungsri, Khajornpat Boonprasert, Warut Wongkalasin, Pongpon Homkong, Suthathip Dejchaisri, Worawit Wajjwalku, Kulnasan Saikhun

**Affiliations:** 1 Faculty of Veterinary Medicine, Kasetsart University, Nakhonpathom 73140, Thailand; 2 Center for Agricultural Biotechnology, Kasetsart University, Nakhonpathom 73140, Thailand; 3 The National Elephant Institute, The Forest Industry Organization, Lampang 52190, Thailand; 4 Faculty of Veterinary Medicine, Chiangmai University, Chiangmai 50100, Thailand; 5 Maesa Elephant Camp, Chiangmai 50000, Thailand; 6 Institute of Molecular Biosciences, Mahidol University, Nakhonpathom 73170, Thailand

## Abstract

**Background:**

Artificial insemination (AI) using frozen-thawed semen is well established and routinely used for breeding in various mammalian species. However, there is no report of the birth of elephant calves following AI with frozen-thawed semen. The objective of the present study was to investigate the fertilizing ability of chilled and frozen-thawed semen in the Asian elephant following artificial insemination (AI).

**Methods:**

Semen samples were collected by from 8 bulls (age range, 12-to 42-years) by manual stimulation. Semen with high quality were either cooled to 4°C or frozen in liquid nitrogen (-196°C) before being used for AI. Blood samples collected from ten elephant females (age range, 12-to 52-years) were assessed for estrus cycle and elephants with normal cycling were used for AI. Artificial insemination series were conducted during 2003 to 2008; 55 and 2 AI trials were conducted using frozen-thawed and chilled semen, respectively. Pregnancy was detected using transrectal ultrasonography and serum progestagen measurement.

**Results:**

One female (Khod) inseminated with chilled semen became pregnant and gave birth in 2007. The gestation length was 663 days and the sex of the elephant calf was male. One female (Sao) inseminated with frozen-thawed semen showed signs of pregnancy by increasing progestagen levels and a fetus was observed for 5 months by transrectal ultrasonography.

**Conclusion:**

This is the first report showing pregnancy following AI with frozen-thawed semen in the Asian elephant. Successful AI in the Asian elephant using either chilled or frozen-thawed semen is a stepping stone towards applying this technology for genetic improvement of the elephant population.

## Background

Although the Asian elephant has been listed in appendix I of the Convention International Trade in Endangered Species (CITES) since 1972, the population throughout Asia still is declining due to habitat loss and poaching. As the number of wild elephants continues to decline, maintaining healthy captive populations is vital. Because of difficulties in transporting a bull elephant from one institution to another for natural breeding, artificial insemination (AI) is an alternative tool for genetic management in this species. Although elephant calves have been produced following AI using either fresh or chilled semen [1-3] there has been no report of the birth of elephant calves after AI with frozen-thawed semen. Our previous studies showed that acceptable semen quality can be obtained after freezing and thawing [4,5]; however, pregnancy was not established after AI [6], indicating a need to develop an effective cryopreservation technique for Asian elephant spermatozoa.

Two AI techniques in elephants have been reported; i.e. surgical AI via vestibulotomy incision [1] and endoscopic AI [2,3]. Both techniques are impractical in Thailand. Surgical AI is not suitable to use due to the complication of wound infection and concerns for animal welfare, whereas endoscopic AI requires special accessory equipment: a balloon catheter to distend the reproductive tract [1]. Therefore, the objective of this study was to investigate the fertilizing ability of either chilled or frozen-thawed semen using the modified endoscopic AI technique described herein.

## Methods

### Chemicals

All chemicals in this study were purchased from Sigma Chemical Company (Sigma, St. Louis, MO, USA) unless stated otherwise.

### Animals

All animals and the experiments were approved by the National Elephant Institute. Eight elephant bulls (age range, 12-to 42-years-old) housed at the National Elephant Institute, Forest Industry Organization, Lampang, Thailand were used as semen donors. Ten elephant females (age range, 12-to 52-years) were used for AI. The elephants were fed with grass, banana and sugar cane during the day and allowed to roam in the jungle at night.

### Semen collection and evaluation

Semen samples were collected by manual manipulation as described by Schmitt and Hildebrandt [7]. Each ejaculate was immediately evaluated for volume, sperm concentration, progressive motility, sperm viability and pH [8]. Sperm concentration was assessed using a haemocytometer. Progressive motility was assessed visually under a phase-contrast microscopy by two independent technicians. Samples containing ≥80% motile spermatozoa, and ≥50 × 10^6 ^cells/ml were used in this study.

### Preparation of frozen-thawed semen

Semen samples collected from 6 bulls (Dindum, Pamae, Yuak, Koong, Tadaeng and Jumpui) were frozen by the method as previously described [4]. Briefly, fresh semen was diluted with TEST extender (1:1) at room temperature. TEST extender consisted of 5.54% Tes [N-tris(hydroxymethyl)methyl-2-aminoethane-sulphonic acid], 1.15% Tris-(hydroxymethyl)-aminomethane, 0.4% glucose and 20% egg yolk. The semen mixture was cooled from room temperature (28 to 32°C) to 5°C at a rate of 1°C/min using the Biocool II cooling machine (FTS^® ^Systems, Inc., Stone Ridge, NY, USA). The semen mixture was then maintained at 5°C on ice or in the cooling machine. Thereafter, equal volume of TEST extender containing 10% glycerol was divided into four parts and each part was added to the semen mixture every 15 min and further equilibrated at 5°C for 1 h. The equilibrated mixture was packed in a 0.5 ml labeled plastic straw container (Kruuse, Ltd., Leeds, UK) by manual technique. The straws were sealed with sealing powder (ARSTM, Chino, CA, USA) kept in plastic tubes on ice, and placed on a stainless steel rack in a styrofoam box containing liquid nitrogen. The straws were held at 2.5 cm above liquid nitrogen level for 10 min and then were plunged into liquid nitrogen until use for AI.

The frozen samples were rapidly thawed by placing the straws in a 37°C water bath for 30 s. The semen samples were expelled into a 50 ml tube and kept in a 37°C water. Ten microlitres of the thawed semen were then taken and placed on a pre-warmed microscopic slide and the progressive motility of post-thaw spermatozoa was assessed under light microscopy. The frozen-thawed samples containing ≥40% motile spermatozoa were used for AI.

### Preparation of chilled semen

Semen samples collected from 2 bulls (Japatee and Sai) were prepared and used for AI as chilled semen. Fresh semen was diluted with TEST extender [4] to a final volume of 150 ml. The semen mixture was cooled from room temperature (28–32°C) to 4°C at a rate of 1°C/min using the Biocool II cooling machine (FTS^® ^Systems, Inc., Stone Ridge, NY, USA). The semen mixture was then maintained at 4°C on ice or in the cooling machine and warmed at 37°C before being used for AI.

### Hormonal assay for determination of estrous cycle

A 10-ml blood sample was collected from an ear vein weekly for hormonal assay. When progestagen concentrations decreased to baseline (i.e., during the follicular phase), blood was collected daily (at the same time each day) until concentrations increased again. Blood samples were allowed to clot at room temperature for 1 to 2 h, and then centrifuged at 1500 g for 5 min to separate serum from blood cells. Serum samples were transferred into 1.5 ml microtubes and stored at -20°C until analysis.

### Serum progestagens

Serum progestagens were analyzed using enzyme immunoassay (EIA) as previously described [9]. This EIA employed a monoclonal antibody against progesterone (1:10,000; Quidel clone #425 Quidel clone#425; supplied by C. Munro, Davis, CA, USA) that has been shown to cross-react with a variety of reduced progesterones in the serum and excreta of a wide range of species, including elephants [10]. Bound progesterone was visualized using a horseradish peroxidase-conjugated antibody to progesterone (1:40,000; C. Munro, University of California-Davis, USA) and quantification was achieved by comparing the level of colour development to that for known progesterone standards using an ELISA plate reader (TECAN^® ^sunrise absorbance reader; Tecan Austria GmbH, Austria). The sensitivity of the assay was 0.1 ng/ml and the inter-assay coefficients of variation (CV) for high and low concentration controls, and the intra-assay coefficients of variation (CV) for high and low concentration controls were 12.1%, 13.0%, 3.9% and 8.6% respectively.

### Serum luteinizing hormone (LH)

Serum luteinizing hormone (LH) was analyzed using EIA as previously described [9]. The LH EIA utilized a monoclonal anti-bovine LH antiserum (518-B7), a biotin-conjugated ovine LH label, and bovine LH (NIH-LH-B10; AFP-5551B) standards [11]. This monoclonal antibody was validated and approved to detect LH in Asian elephants [3,11]. The LH label was prepared using an EZ-LinkTM Sulfo-NHS-LC-Biotinylation kit (Pierce, Rockford, IL, USA). Sensitivity of the assay was 0.16 ng/mL. The inter-assay coefficient of variation (CV) for the high concentration control was 7.60% and for the low control was 8.47%.

### Artificial insemination

Artificial insemination series were conducted during 2003 to 2008. Fifty five and two AI attempts were conducted with frozen-thawed and chilled semen in six and two elephant females, respectively. Three inseminations (once per day) per series were performed on days -1, 0, 1 of the ovulatory LH surge [3]. Fifty milliliter of semen was used per inseminations. The female was standing immobilized using 0.04 mg/kg Xylazine HCL (RomazineR 100; Jurox Pty. Ltd., Rutherford, NSW, AUS) intravenously [12].

**Figure 1:**
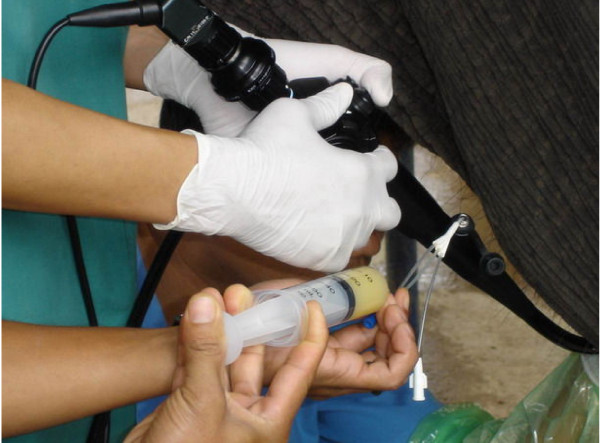
**Semen was deposited via insemination catheter through endoscope working channel**.

For AI, the endoscopic technique was modified by increasing inflated air from external electric air pump without using the balloon catheter. The inflated air could enlarge the vestibular, vaginal and cervical canal. After the tip of endoscope reached and fixed at the cervical canal, the air tube was removed and replaced by insemination catheter (IC). The IC was passed through the working channel of the endoscope and penetrated deep into the body of uterus. The semen was deposited into body of uterus without signs of semen reflux (Figure 1). A fiber optic video endoscope, video processing circuit and light source of PENTAX (EPM3300 and EC3830 fz) were used. Transrectal ultrasonography (ALOKA; SSD 500 with convex transducer probe; 3.5 MHz and linear transducer; 5.0 MHz and Sonosite model 180 Plus hand-carried ultrasound with convex transducer probe C60 2.0–5.0 MHz) was used to guide the endoscope and to locate the site of semen deposit.

### Pregnancy detection

Pregnancy diagnosis was performed by serum progestagen measurement and transrectal ultrasonography. Blood samples were collected weekly and serum progestagens were assessed by EIA as described above [3]. Transrectal ultrasonography was performed weekly between 2 and 4 months after AI as previously described [1].

### Parentage testing

DNA templates were isolated from whole blood of male, female and AI calves using high Pure PCR Template Preparation Kit (Roche Diagnostics, Basel, Switzerland). Five primer pairs of microsatellite markers; LA2 [13], EMX-1 [14], LafMS03 and LafMS05 [15] and FH 94 [16] were used for PCR [17].

Extracted DNA was amplified in 25 μl reaction volumes containing 2.5 μl Supertaq 10× buffer (Applied Biosystems, Foster city, CA, USA), 5 mM dNTP (1 μl) (Qiagen Inc., Valencia, CA, USA), 5 pmol primers (1 μl), 0.4 U of Super taq DNA polymerase (0.25 μl) (Applied Biosystems, Foster city, CA, USA), 50 ng of extracted DNA (1 μl) and Milli Q water (18.25 μl). PCRs were performed in Peltier Thermal Cycler 220 (M J Research Inc., Waltham, MA, USA). PCR consisted of a single denaturation step at 94°C for 2 min followed by a cycle and 34 cycles of 94°C denaturation for 30 s, 30 s of primer annealing with specific temperature of each primer, 30 s of primer extension at 72°C followed by a single extension of 72°C for 2 min.

Positive PCR products generated by specific primers of microsatellite markers were fractionated on a 1.5% agarose gel (SEAKEM^® ^LE agarose, BioWhittaker Molecular Applications, Rockland, ME, USA). Then, all PCR products were genotyped on a Beckman Coulter sequencer using WellRED™ primers (Proligo) and fragment analysis software version 5.0 (Beckman Coulter). Parentage analysis was performed by parentage exclusion method [18].

## Results

### Semen collection and preparation

A total of 24 ejaculates obtained from six bulls were prepared as frozen semen. Data on semen quality of fresh, chilled and frozen-thawed samples are shown in Table 1. Frozen-thawed samples containing spermatozoa with >40% motility achieved from 4 bulls (Dindum, Pamae, Tadaeng and Jumpui) were used for AI, whereas samples containing spermatozoa with <40% motility achieved from 2 bulls (Yuak and Koong) were discarded. Sperm motility of frozen-thawed samples used for AI was ranged from 40 to 50%. The average sperm motility of frozen-thawed semen was 45.5 ± 14.2%. In case of chilled semen, 2 ejaculates collected from 2 bulls (Japatee and Sai) were cooled to 4°C and maintained for 3 days for AI. During preservation for up to 3 days, the average sperm motility of chilled semen was 80 ± 35, 60 ± 24 and 50 ± 15% on the first, second and third day of insemination, respectively. A progressive reduction of the percentage of viable sperm was observed.

**Table 1:** Mean ± SD of semen quality of fresh, chilled and frozen-thawed samples.

	Semen samples				
Parameters	Fresh	Chilled			Frozen
		Day 1	Day 2	Day 2	
Volume (ml)	31.0 ± 31.3	-	-	-	-
pH	7.6 ± 0.7	-	-	-	-
Concent. (×10^6^/ml)	887.6 ± 487.1	208.0	208.0	208.0	100.0
Motility (%)	82.7 ± 3.6	80.0 ± 35.0	60.0 ± 24.0	50.0 ± 15.0	45.5 ± 14.2
Viable sperm (%)	76.8 ± 14.4	71.5 ± 21.3	33.3 ± 10.2	20.0 ± 8.4	50.9 ± 13.6

### Hormonal assay for determination of estrous cycle

Eight out of 10 elephants assessed for estrus cycle showed normal cycling and were selected for AI. The average estrous cycle length was 4 months, with ~3 months for luteal phase and ~1 month for follicular phase. The onset of the luteal phase was defined as the first point at which serum progestagen concentrations increased above 0.3 ng/ml and then retained high levels (1 to 2 ng/ml) for at least 2 weeks. At the end of luteal phase, serum progestagen concentrations started to decline and reached the baseline (0.1 ng/ml) within 1 to 2 weeks and then remained at that level for about 4 weeks.

### Pregnancy detection

**Figure 2:**
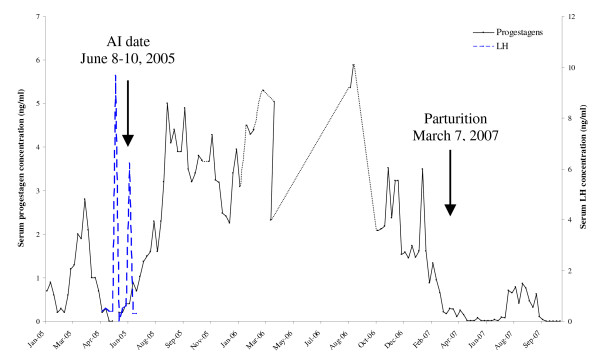
**Serum progestagens and LH concentrations (ng/ml) of the female elephant which was inseminated with chilled semen**. The AI and parturition date confirmed pregnancy of 663 days. Discontinuous lines represent missing data due to blood samples could not be collected.

**Figure 3:**
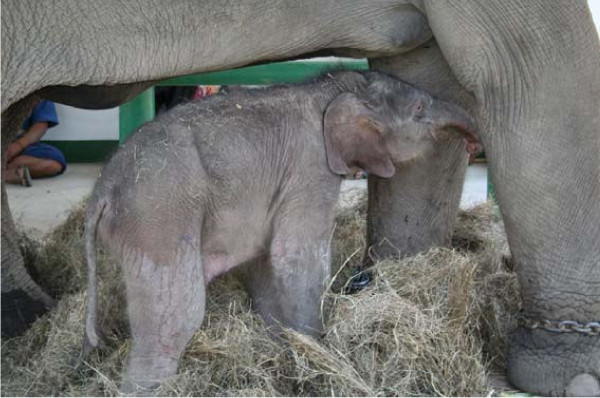
**AI baby born from chilled semen with his mother**.

In case of AI with chilled semen, one out of two females, namely "Khod", showed progressive increase of serum progestagens after AI (Figure 2). The level of serum progestagen concentrations reached 2 ng/ml within 2 months after AI (June, 2005) and a high level of progestagen (2–5 ng/ml) was retained for more than 20 weeks, which indicating pregnancy. Serum progestagen started to decline to the baseline one month before birth (Figure 2). Transrectal ultrasonography of pregnant cow showed enlargement of the uterine wall at 2 months after AI. An increase of uterine lumen that filled with clear fluid was found between 3 and 4 months. We were unable to find a fetus between 2 and 4 months after AI. Some changes in Khod's behavior during pregnancy period were observed. Khod's anatomy was not remarkably changed during pregnancy period except that her belly was enlarged during the last sixth month period. Furthermore, one month before calving, her breasts were enlarged and had a clear secretion released when they were stimulated. Khod gave a birth on March 7, 2007 with birth weight of 51 kg. The gestation length was 663 days and the sex of elephant baby was male (Figure 3). The calf was apparently normal in health and behaviour and had a normal suckling reflex.

**Figure 4:**
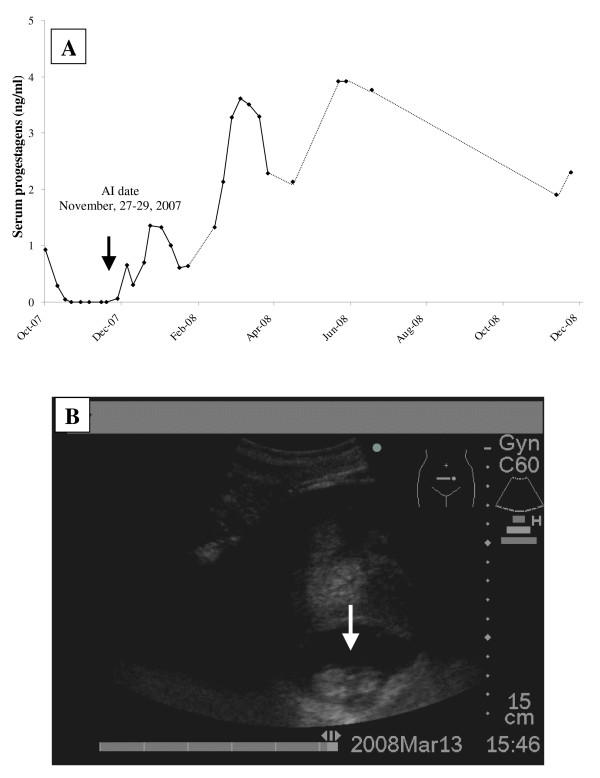
**A) Serum progestagens (ng/ml) of the female elephant which was inseminated with frozen semen**. Increase and remain of serum progestagens at high level more than 20 weeks after AI indicated pregnancy. Discontinuous lines represent missing data due to blood samples could not be collected. B) Sonogram of elephant conception at day 107 post ovulation. The white arrow indicated embryo with the diameter of 43 mm.

In case of AI with frozen-thawed semen, one out of 6 females namely "Sao" showed progressive increase of serum progestagen to a level of 2–8 ng/ml (Figure 4A). Transrectal ultrasonography showed enlargement of the uterine body and a fetus was observed during 5 months of pregnancy (Figure 4B). The pregnant cow is an ongoing pregnancy.

### Parentage testing

Microsatellite profiles of AI elephant and his parents (Japatee and Khod) are shown in Table 2. The results revealed the Medelian inheritance of all markers. They indicated that the AI calf is the son of Japatee and Khod.

**Table 2:** Size of microsatellite markers of AI baby family

		Loci				
Elephant	Status	LA2 (bp)	EMX1 (bp)	FH94 (bp)	LafMS03 (bp)	LafMS05 (bp)
Japatee	Father	223/229	146/150	220/224	146/152	142/150
Khod	Mother	229/229	134/150	220/222	138/146	142/150
AI baby	Child	223/229	146/150	220/222	138/152	142/150

## Discussion

The present study demonstrated that AI with a modified endoscopic technique using chilled semen was successfully used to produce a elephant calf. The modified endoscopic technique using frozen-thawed semen demonstrated establishment of pregnancy. To our knowledge this is the first paper reporting establishment of pregnancy with AI using frozen-thawed semen in the Asian elephant. This suggests that AI may present an alternative approach to traditional breeding for genetic and management in this species.

The elephant is an important animal and is recognized as the symbol of Thailand. Although the elephant is highly valued and plays an important economic role in our country the population of captive and wild elephants has decreased gradually. It is well accepted that a major cause for the decline of captive elephants in Thailand is the loss of opportunity for propagation caused by the practice of separating male and female elephants and distributing them to either elephant camps or zoos for tourism purposes. Furthermore, the difficulty and cost in transporting elephant bulls for traditional breeding and the long period of pregnancy (~2 year) causes elephant owners to ignore breeding programs. In addition to natural breeding, AI with either chilled or frozen-thawed semen will provide genetic diversity. Moreover, ovLH surge, which induced ovulation, induction protocol was developed [19]. This novel method is an important advance for AI [20], and is useful for reducing the labor and number of samples needed for identifying the anLH surge to time the assisted breeding 3 weeks later [3].

Although the present study successfully produced one elephant calf out of 2 AI trials using chilled semen the success rate of AI with frozen-thawed semen is extremely low (one successful pregnancy out of 55 AI trials). The reason for the low success rate of AI with frozen-thawed semen samples is not clear. It might be due to the low sperm quality following freezing and thawing compared to chilled and fresh semen. High quality of post-thaw sperm is a prerequisite for the success rate after AI. During freezing and thawing of elephant semen changes in the traditional sperm parameters, including sperm motility, viability, acrosome integrity and mitochondrial activity have been detected [4,5]. Furthermore, ultrastructural investigations using transmission electron microscopy (TEM) confirmed that the freezing and thawing procedure caused structural damage to elephant sperm, especially in the plasma membrane, acrosome and mitochondria. These changes may cause the low success rate of AI with frozen-thawed semen. In addition, other factors could affect the success of AI such as detection of the LH surge, AI technique, health status and the environment. Further studies revealing how these factors affect the success of AI using frozen-thawed semen are needed.

The endoscopic AI technique in this present study was modified from the previous report by Hildebrandt et al [1]. We increased the inflating ability of the reproductive tract by connecting the air from an external electric pump to the working channel of the endoscope. The increasing air pressure enlarged the vaginal canal, slightly opened the cervical opening and allowed the easier insertion of IC deep into the cervical canal. The IC passing through the working channel of the endoscope penetrated deep inside the cervical canal and the deposition of semen into this position prevented semen reflux. The increased air pressure from an external source thus benefitted the procedure. It did not require use of a special balloon catheter to distend the reproductive tract. Khod is a primiparous elephant. The anatomy of the reproductive tract in the female elephant changes after having offspring. In the nulliparous elephant, the hymen is maintained and its opening diameter is very narrow. In a primiparous elephant, the hymen and two blind pouches between vestibule and vagina disappear [1]. This might be a key point of success and failure in previous AI attempts in nulliparous elephants (unpublished data). Thus, the adjustment of air power for increasing the ability to inflate the hymen opening in nulliparous elephant may be tried in the future.

Early pregnancy could not be detected by physical examination, therefore, the unknown pregnant cow is normally used in the routine work without proper management, which can lead to dystocia from an oversized fetus [21] or to abortion; thus, it is crucial to diagnose pregnancy in elephants for better management. Pregnancy and parturition diagnosis can be performed by reproductive endocrine monitoring. Serum progestagens decline and drop to baseline 2 to 5 days before parturition [12,22-24]. In agreement with those reports, serum progestagens of Khod dropped to baseline 2 days before giving a birth, which was useful for managing parturition as previous described. Daily monitoring of serum progestagens during the last two weeks of pregnancy was useful for parturition management.

In this present study, all of five microsatellite markers were amplified from all members in the AI family. In agreement with our previous study, cross-species amplification was observed for LA2, LafMS03, LafMS05 and FH 94 primer pairs [17]. For the last primer, LafMS05, AI baby and his parents have a same size of genotype of both alleles. It showed a monomorphism in this family. However, the parentage testing with these five microsatellite markers analysis revealed the relationship between members in the family. Therefore, it can be stated that the AI baby born with chilled semen is the son of Japatee and Khod.

## Conclusion

Our success in producing an elephant calf with chilled semen and establishment of pregnancy with frozen-thawed semen confirmed that AI could be used as an alternative approach for breeding management of this endangered species.

## Competing interests

The authors declare that they have no competing interests.

## Authors' contributions

NT and KS designed the study and contributed to the writing the manuscript. AP and WW contributed to the analysis and discussion of data. CT and PP participated in hormonal analysis. SM, KK, TA, SJ, RR, KB, WW, PH and SD participated in semen collection and artificial insemination. All authors read and approved the final manuscript.
